# Common *FLG* Mutation K4671X Not Associated with Atopic Dermatitis in Han Chinese in a Family Association Study

**DOI:** 10.1371/journal.pone.0049158

**Published:** 2012-11-13

**Authors:** Ruhong Cheng, Ming Li, Hui Zhang, Yifeng Guo, Xilan Chen, Jianfeng Tao, Aifang Jiang, Jiecheng Gan, Huaishan Qi, Hong Yu, Wanqing Liao, Zhirong Yao

**Affiliations:** 1 Department of Dermatology, Xinhua Hospital, Shanghai Jiaotong University School of Medicine, Shanghai, China; 2 Department of Dermatology, Changzheng Hospital, The Second Military Medical University, Shanghai, China; CNRS-University of Toulouse, France

## Abstract

**Background:**

Filaggrin gene (*FLG*) mutations have been identified as the cause of ichthyosis vulgaris (IV) and major predisposing factors for atopic dermatitis (AD). The relationship among AD, IV and *FLG* mutations has not been clarified yet. Mutations 3321delA and K4671X, two of the most common mutations in Chinese patients, were both statistically associated with AD in case-control studies.

**Materials and Methods:**

A group of 100 family trios (a total of 300 members with one affected AD proband and both parents) were recruited and screened for three filaggrin null mutations (3222del4, 3321delA and K4671X). The subjects’ manifestations of AD and IV were assessed by two experienced dermatologists and recorded in detail. The relationship of common mutations to AD were assessed using both case-control and family-based tests of association. Filaggrin expression was measured in skin of 3 subjects with K4671X heterozygote and the normal control using quantitative real-time RT-PCR and immunohistochemistry.

**Results:**

Of 100 probands for AD, 22 were carriers for common *FLG* mutations and only 2 of them were from 40 none-IV family trios (5.00%), consistent with that of the healthy control group (3.99%, P>0.05). Significant statistical associations were revealed between AD and 3321delA (P<0.001, odds ratio 12.28, 95% confidence interval 3.35–44.98) as well as K4671X (P = 0.002, odds ratio 4.53, 95% confidence interval 1.77–11.60). The family-based approach revealed that 3321delA was over-transmitted to AD offspring from parents (T:U = 12∶1, P = 0.003) but failed to demonstrate transmission disequilibrium between K4671X and AD (T:U = 10∶8, P = 0.815). Moreover, compared to the normal control, filaggrin expression at both mRNA and protein levels in epidermis of subjects with K4671X^heter^ was not reduced.

**Conclusions:**

AD patients from none-IV family trios have low probability of carrying *FLG* mutations. The present family samples confirmed the susceptibility of mutation 3321delA to AD in Han Chinese. K4671X was not a pathogenic mutation.

## Introduction

Atopic dermatitis (AD) is characterized by skin dryness and chronic inflammation [1]. Ichthyosis vulgaris (IV, OMIM #146700) is the most common inherited disorder of keratinization, exhibiting palmar hyperlinearity, keratosis pilaris, and a fine scale that is prominent over the lower abdomen, arms, and legs [2], [3]. Previous reports have shown that 2.5% to 37% of patients with AD have clinical evidence of IV [4]. Between 37% and 70% of patients with IV display clinical features of AD [5]. Mutations in the filaggrin gene (*FLG*), the gene encoding *profilaggrin/filaggrin*, have been identified as the underlying cause of IV and shown to predispose patients to AD [2], [6]. However, the relationship among AD, IV and *FLG* mutations has not been clarified yet.

Common *FLG* mutations in Europeans, R501X and 2282del4 in repeat 1 of exon 3, were demonstrated to be associated with AD both by case-control study and family-based analysis [7]. *FLG* null variants were also found to have strong association with AD among the Chinese. The associations of common mutations in Chinese, 3321delA and K4671X, with AD were both statistically significant in case-control studies [8], [9], however, have not been evaluated in family-based association test.

Analysis of available detailed *FLG* genotype information gathered from a collection of carefully phenotyped family trios provides a useful tool to help investigate the relationship among *FLG* mutations, AD, and IV as well as to reassess the association between *FLG* mutations and AD by family-based association test.

## Results

### Clinical Features

The clinical characteristics of the family trios were shown in Table 1. The average age of 100 AD probands was 2.87±3.04 years compared with 13.90±4.37 years of the 301 controls (104 girls and 197 boys without AD or IV) [9]. In 100 AD trios, there were 40 none-IV family trios(none of the AD probands and their parents were presented with the IV phenotype). The normal control was a 34-year-old man without AD or IV. Patient 1 and 2 were female parents at the age of 33 and 38. They were not presented with AD or IV. Patient 3 was a 26-year-old adult with mild AD but no IV.

**Table 1:** Phenotype characteristics of 100 trios included in the analyses.

Clinical information	Trios(n = 100)	
	Parents(200)	Offspring(100)
Han Chinese, n* (%)	200 (100%)	100 (100%)
Male sex, n* (%)	100 (50%)	66 (66%)
Mean±SD age, y	ND	2.87±3.04
Simple atopic dermatitis, n* (%)	39 (19.5%)	87 (87%)
Isolated ichthyosis vulgaris, n* (%)	53 (26.5%)	–
atopic dermatitis + ichthyosis vulgaris, n* (%)	29 (14.5%)	13 (13%)
Keratosis pilaris, n* (%)	44 (22%)	4 (4%)
Palmar hyperlinearity, n* (%)	72 (36%)	31 (31%)
Cheilitis, n* (%)	22 (11%)	21 (21%)
Dyshidrosis, n* (%)	53 (26.5%)	8 (8%)
Mild atopic dermatitis (SCORAD,0–24points)	ND	20 (20%)
Moderate atopic dermatitis (SCORAD,25–50points)	ND	46 (46%)
Severe atopic dermatitis (SCORAD,51–103points)	ND	34 (34%)

* Number affected/total number with data available.

SCORAD, SCORing atopic dermatitis.

ND, not done.

### Genotyping

The genotyping success rate was 100%. Analysis of Mendelian inheritance within the families revealed no significant errors. Of 100 AD probands, 22 (22%) were carriers for common *FLG* null alleles, including 20 heterozygous, 1 homozygote for 3321delA, and 1 compound heterozygote for 3321delA and K4671X. Among 200 AD parents, 31 (15.5%) were carriers for common *FLG* null alleles, 30 heterozygous along with 1 compound heterozygote for 3321delA and K4671X. Twenty-three out of 24 *FLG* null alleles carried by 22 probands were inherited from their parents. The patient with a spontaneous mutation K4671X was a 22-month-old boy with moderate AD but no IV phenotype. No homozygous for either K4671X or 3222del4 was observed in the present family cohort. (Table 2) Twelve out of 301 healthy controls were heterozygote carriers for common *FLG* mutations (1 for 3222del4, 3 for 3321delA, and 8 for K4671X).

**Table 2:** Frequencies of filaggrin gene null alleles in parents, offspring as well as normal controls.

Genotype	3321delA			K4671X			3222del4			Combined genotype		
	parents	offspring	control	parents	offspring	control	Parents	offspring	control	parents	offspring	control
**AA**	187(93.5%)	89(89%)	298(99%)	182(91%)	89(89%)	293(97.3%)	199(99.5%)	99(99%)	300(99.7%)	169(85.0%)	78(78%)	289(96%)
**Aa**	13(6.5%)	10(10%)	3(1.0%)	18(9%)	11(11%)	8(2.7%)	1(0.5%)	1(1%)	1(0.3%)	30(14.5%)	20(20%)	12(3.9%)
**aa**	0	1(1%)	0	0	0	0	0	0	0	1(0.5%)	2(2%)	0

AA, Wild-type/wild-type/wild-type *FLG* genotype for 3321delA, 3222del4, and K4671X variants;

Aa, Heterozygous genotype for 3321delA, 3222del4 or K4671X;

aa, Homozygous 3321delA, K4671X or 3222del4 genotype or compound heterozygous genotype.

**Figure 1:**
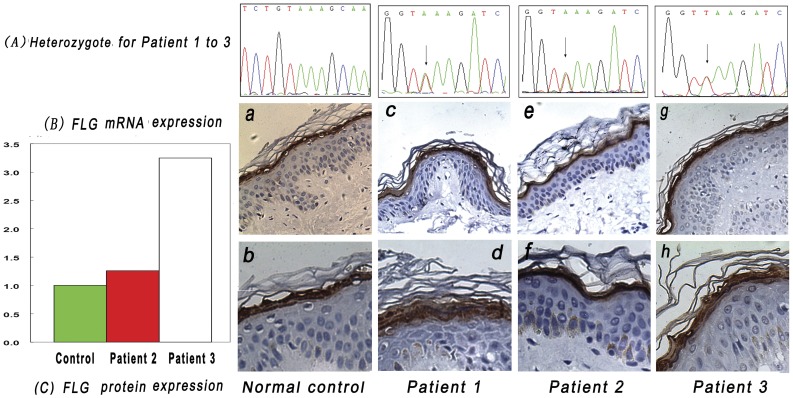
Filaggrin gene mutation analysis and comparison of filaggrin expression. The normal control was an *FLG* ^wt^ male adult. Patient 1 and 2 with K4671X^heter^ variant were mothers of 2 atopic dermatitis (AD) patients. Patient 3 was an adult male AD patient with K4671X^heter^ variant. Skin with normal appearance was got from the 4 subjects. (A) Identification of *FLG* mutations in 3 patients. Patient 1, 2, and 3 were all heterozygote for K4671X. (B) Quantitative Real-time reverse transcription–polymerase chain reaction analysis of *FLG* mRNA expression in the skin of normal control and 2 patients with K4671X^heter^ variant. Direct sequence analysis of *FLG* cDNA from mRNA expressed in skin samples further confirmed that Patient 2 and 3 were heterozygote for K4671X. *FLG* mRNA expression was not reduced either in Patient 2 or 3: *FLG* mRNA expression in Patient 2/3: *FLG* mRNA expression in control was1.26 and 3.25, respectively. (C) Immunohistochemical staining using antifilaggrin monoclonal antibody in 4 subjects at the same time (a,c,e,g,i: Original magnification×100; b,d,f,h,j:Original magnification×200). Immunohistochemically, it was obvious that filaggrin was strongly positive in the epidermis of Patient 1, 2, and 3(c–h).

No *FLG* mutation was found in the normal control. Patient 1, 2, and 3 were heterozygote for *FLG* mutation K4671X.(Figure1A)In addition, mutation K4671X was identified in cDNA of the skin from Patient 2 and 3.

### Relationship Among AD, IV and *FLG* Mutations

Of 22 probands carrying *FLG* null alleles, 14 heterozygote carriers (7 for 3321delA and 7 for K4671X) did not exhibit the IV phenotype. But it was noted that 20 (33.33%) probands with common *FLG* mutations were from 60 families with at least one parent affected by IV whereas only 2 (5.00%) were from 40 none-IV family trios (P = 0.001, OR = 9.50, 95%CI:2.08–43.43), consistent with that of the healthy control group (3.99%, P>0.05). (Table 3).

**Table 3:** Prevalance and comparison of compound genotype for common *FLG* mutations in various groups.

Probands and controls	Compound genotype for common *FLG* mutations			
	n(n%)	P-value	OR	95%CI
control (301)	12(3.99%)	–	–	–
All probands(100)	22(22.00%)	<0.001	6.79	3.22–14.33
Simple AD (87)	14(16.09%)	<0.001	4.62	2.05–10.41
AD with IV(13)	8(61.50%)	<0.001	38.53	10.96–135.54
AD from none-IV Family trios (40)	2(5.00%)	>0.05	–	–
AD from families with parental IV (60)	20(33.33%)	<0.001	12.04	5.47–26.49

*FLG*, filaggrin gene; AD, atopic dermatitis; IV, ichthyosis vulgaris.

OR: odds ratio; CI : confidence interval;

### Relationship of Common *FLG* Mutations with AD-associated Phenotype as Well as the SCORAD

The compound genotypes for common *FLG* variants were associated with three co-existing phenotype, IV, keratosis pilaris, and palmar hyperlinearity. (Table 4) However, the compound genotypes for common *FLG* variants were not associated with the total SCORAD (46.2±18.8 vs. 39.6±17.7, P = 0.127) in an independent-samples T test.

**Table 4:** Analysis of associations between combined common filaggrin gene mutations and atopic dermatitis associated phenotype.

AD probands	Combined *FLG* genotype		
	AA	Aa/aa	P
AD with IV	5	8	0.001
AD without IV	73	14	
AD with Palmar hyperlinearity	16	15	<0.001
AD without Palmar hyperlinearity	61	8	
AD with Keratosis pilaris	0	4	0.002
AD without Keratosis pilaris	78	18	
AD with Dyshidrosis	7	1	0.817
AD without Dyshidrosis	71	21	
AD with Cheilitis	14	7	0.265
AD without Cheilitis	64	15	
AD with Infra-auricular and retroauricular fissuring	20	3	0.237
AD without Infra-auricular and retroauricular fissuring	58	19	

*FLG*, filaggrin gene; AD, atopic dermatitis; IV, ichthyosis vulgaris.

### Family-based Association Test

Significant statistical associations were revealed between AD and 3321delA (P<0.001, odds ratio 12.28, 95% confidence interval 3.35–44.98) as well as K4671X (P = 0.002, odds ratio 4.53, 95% confidence interval 1.77–11.60). Family-based approach revealed that 3321delA was over-transmitted to AD offspring from parents (T:U = 12∶1, P = 0.003) but failed to demonstrate transmission disequilibrium between K4671X and AD (T:U = 10∶8, P = 0.815) (Table 5).

**Table 5:** Association analysis of filaggrin mutations with atopic dermatitis in both family and case-control studies.

Results	3321delA		K4671X		3222del4	
	Case-control	family	Case-control	family	Case-control	family
P value	<0.001	0.003	0.002	0.815	0.437	–
T:U	–	12∶1	–	10∶8	–	–
Odds Ratio	12.28	–	4.53	–	–	–
95% CI	3.35–44.98	–	1.77–11.60	–	–	–
n%(100 probands)	11.0%	–	11.0%	–	1.0%	–
n%(301 controls)	1.0%	–	2.7%	–	0.3%	–

P value represented as ratio of transmitted:untransmitted (T:U) filaggrin gene minor alleles;

CI : confidence interval.

### Quantitative Real-time RT-PCR Analysis and Immunohistochemistry

*FLG* mRNA expression was not reduced in both Patient 2 and 3 in real-time RT-PCR analysis. (Figure 1 B) Immunohistochemical staining revealed that profilaggrin /filaggrin peptides were also not reduced in the epidermis of Patient 1, 2 and 3. (Figure 1C).

## Discussion

In 2006, Smith et al. succeeded in demonstrating that loss-of-function mutations encoding *FLG* cause IV in an incomplete-dominant pattern. Palmer et al. conducted a further research using the 15 families studied for the IV research and presented a new and striking theory that AD was inherited as an incomplete-dominant trait in these families with high penetrance in *FLG*-null homozygous or compound heterozygous and reduced penetrance in heterozygous [2], [6], [10]. Because of the incomplete dominant pattern of *FLG* gene in the pathogenesis of IV, it is conceivable that in our previous and current studies of 361 AD cases in total, 82 cases without the IV phenotype were carriers for common *FLG* mutations [11]. However, in the current study of 100 AD family trios, 40 were none-IV families, from which only 2 AD probands were common *FLG* mutation carriers (5%). It was noted that the mutation rate of combined *FLG* mutations among these 40 probands was consistent with that of healthy control group (P>0.05). The data meant a convenient method for initial exclusion of AD patients without *FLG* mutations in clinic. In addition, *FLG* mutations were associated with palmar hyperlinearity and keratosis pilaris significantly. However, the severity of AD was not correlated with the *FLG* genotype, which has been demonstrated in our previous studies [8], [9].

In the current study, we found that common *FLG* mutation 3321delA, located in repeat 2 of exon 3, was associated with AD both in the case-control and family-based studies. Therefore, the association of 3321delA with AD is further confirmed. The association test is not available for mutation 3222del4 as only one parent and a single proband are mutation carriers.

Unlike other mutations, such as 3321delA, R501X and 2282del4, mutation K4671X, primarily identified in the Japanese population as p.Lys4021X, is located in the C-terminal incomplete filaggrin repeat [12]. The current case-control study also demonstrated a significant association between K4671X and AD, consistent with our previous studies [8], [9]. But an opposite result was produced from the family-based study. In order to further understand the effect of K4671X on skin barrier function, filaggrin expression in the skin with K4671X^heter^ is required. Previous immunohistochemical staining performed by Nemoto-Hasebe I et al. on two AD patients bearing p.Lys4021X showed profilaggrin/filaggrin peptides were remarkably reduced in the patients’ epidermis but real-time RT-PCR analysis revealed that mRNA expression of *FLG* was not reduced significantly [12], indicating that factors other than *FLG* mutations may lead to filaggrin deficiency in the epidermis of patients bearing mutation K4671X. Experimental evidence for such genetic and environmental modulation includes the demonstration that cytokines from Th2 cells can down-regulate filaggrin expression in AD skin [13]. To minimize factors other than *FLG* mutations on filaggrin exression, we studied the filaggrin expression in skin with normal appearace in subjects with mutation K4671X. The results directly showed that filaggrin expression either at mRNA or protein level was not reduced in the epidemis of subjects with mutation K4671X.

Unlike mutation 3321delA, mutation K4671X could not be found in Taiwanese or Singaporean Chinese AD and IV patients [5], [14]. The paradoxical results from case-control and family studies were mainly attributed to population stratification, the presence of a systematic difference in allele frequencies between subpopulations in a population possibly due to different ancestries. Family-based association test is known to be immune to population stratification because this method uses parental genotype data to control for this spurious admixture, which could not be avoided in case-control designs [15], [16].

In conclusion, AD patients from none-IV family trios have low probability of carrying *FLG* mutations. The present family samples confirmed the susceptibility of mutation 3321delA to AD in Han Chinese but failed to support the same function for mutation K4671X. The filaggrin expression is influenced by multiple factors. Our investigation of filaggrin expression at both mRNA and protein levels demonstrated that K4671X was not a pathogenic mutation.

## Materials and Methods

### Ethics Statement

The present study was approved by the Medical Ethics Committees of the Shanghai Jiaotong University School of Medicine, China. Written informed consents were given by all the adult participants and carers on the behalf of children participants before enrollment in the study. The present study was conducted in accordance with the principles of the Declaration of Helsinki.

### Study Population

A total of 100 unrelated family trios with AD (a total of 300 members with one affected AD proband and both parents) who met the AD criteria of Hanifin and Rajka [17] were recruited. These patients were referred to outpatient dermatological clinics at the Department of Dermatology, Xinhua Hospital affiliated to Shanghai Jiaotong University School of Medicine, China. AD and IV of all 300 subjects were clinically diagnosed by two experienced dermatologists, who also performed a thorough clinical examination and recorded a complete medical history using a standardized questionnaire. All the 100 AD probands also received an overall AD severity grade by SCORAD [18]. The diagnosis of IV was established from clinical features of variable scaling on the extremities, dry skin, palmoplantar hyperlinearity and keratosis pilaris. The DNA samples of 300 subjects were collected. To compare the frequency of *FLG* mutations, DNA samples from 301 normal healthy, unrelated individuals without AD or IV were used as control [9]. In addition, a male adult without AD or IV was selected as the normal control. Two mothers of probands and one male AD patient with mutation K4671X were defined as Patient 1, 2 and 3, respectively. All enrolled individuals were of Chinese Han ancestry.

### Skin Biopsy

Skin biopsy was got from the waist of the normal control, the lateral thigh of Patient 1, the medial upper arm of Patient 2 and the abdomen of Patient 3. The skin for biopsy was normal in appearance without any lesions. The amount of skin from the normal control, Patient 2 and 3 was enough for both quantitative real-time RT-PCR and immunohistochemistry. However, skin from Patient 1 was only prepared for immunohistochemistry.

### *FLG* Genotyping

Genomic DNA samples were extracted from peripheral whole blood using TIANamp Blood DNA kits (TIANGEN Biotech, Beijing, China). Using genomic DNA, all the participants in family trios were screened for three *FLG* mutations (3222del4, 3321delA and K4671X) previously identified among the Chinese population[8]–[9] using an overlapping PCR strategy. A comprehensive sequence of *FLG* was done in the normal control, Patient 1, 2, and 3. Total RNA was extracted from skin samples of the normal control, Patient 2 and 3 using Cell culture and Animal tissue Total RNA extraction and preparation Mini Kit (SLnco, England). Direct sequence analysis of *FLG* cDNA from mRNA expressed in skin samples of Patient 2 and 3 was also performed to screen for the K4671X mutation. PCR primers and conditions were previously described by Sandilands et al. [19] The sequencing of PCR products was conducted on an Applied Biosystems 3730 DNA analyzer (ABI incorporation, Carlsbad, California, USA).

### Measurement of mRNA Expression by Quantitative Real-time PCR

The mRNA levels of *FLG* gene were measured by Quantitative Real Time RT-PCR using SYBR Green Realtime PCR Master Mix (Code No:QPK-201, TOYOBO) in a FTC-3000 Real Time PCR machine(Canada, Funglyn). Primer sequences for filaggrin and glyceraldehyde-3-phosphate dehydrogenase were listed in Table 6. All primer sequences were synthesized by Gene Works (Jierui, Shanghai, China). The cycling conditions were at 94°C for 30 s, followed by 40cycles of 20 s at 94°C, 30 s at 61°C and 30 s at 72°C, and finally 1 min at 72°C. Each sample was run in triplicate. The experiments were repeated for three times.

**Table 6:** Primer sequences for filaggrin and glyceraldehyde-3-phosphate dehydrogenase.

Gene name	Primer sequence (5′ to3’)	Amplicon Size
h-*FLG*-F	CAAATCCTGAAGAATCCAGATGAC	126 bp
h-*FLG*-R	TGCTTGAGCCAACTTGAATACC	
h-*GAPDH*-F	TGAAGGTCGGAGTCAACGGA	225 bp
h-*GAPDH*-R	CCTGGAAGATGGTGATGGGAT	

*FLG*, filaggrin gene;

*GAPDH*, glyceraldehyde-3-phosphate dehydrogenase gene.

### Immunohistochemistry

Immunohistochemistry was done using the standard ABC technique. The mouse monoclonal antibody filaggrin of Novocastra Laboratories Ltd (Newcastle upon Tyne NE12 8EW, United Kingdom) in a working dilution of 1∶50 and the high temperature antigen unmasking technique were used according to the manufacturer’s instructions.

### Statistical Analysis

All statistics were analyzed with spss 19.0 software package (SPSS Inc., Chicago, Illinois, USA). Descriptive statistics for quantitative and qualitative values were calculated and given as means ± SD as well as relative frequencies or absolute numbers, respectively. The statistical significance of differences in genotype frequency among analyzed groups and the associations between *FLG* mutations and AD-associated phenotypes were assessed using a Pearson chi-square test, continuity correction, or Fisher’s exact test as appropriate. An independent-samples T test was performed to evaluate the association between compound common *FLG* mutations and the SCORAD. The strength of association was estimated by calculating the odds ratio (OR) with a 95% confidence interval (CI). Evidence of associations with AD was evaluated using the classical TDT by McNemar test implemented in SPSS 19.0. The level of statistical significance was established at α<0.05. Data for *FLG* mRNA expression were analyzed using the comparative CT method.
